# Comparison of tactile, auditory, and visual modality for brain-computer interface use: a case study with a patient in the locked-in state

**DOI:** 10.3389/fnins.2013.00129

**Published:** 2013-07-24

**Authors:** Tobias Kaufmann, Elisa M. Holz, Andrea Kübler

**Affiliations:** Department for Psychology I, Institute for Psychology, University of WürzburgWürzburg, Germany

**Keywords:** brain-computer interface, tactile auditory and visual modality, locked-in syndrome, user-centered design, end-user testing, assistive technology

## Abstract

This paper describes a case study with a patient in the classic locked-in state, who currently has no means of independent communication. Following a user-centered approach, we investigated event-related potentials (ERP) elicited in different modalities for use in brain-computer interface (BCI) systems. Such systems could provide her with an alternative communication channel. To investigate the most viable modality for achieving BCI based communication, classic oddball paradigms (1 rare and 1 frequent stimulus, ratio 1:5) in the visual, auditory and tactile modality were conducted (2 runs per modality). Classifiers were built on one run and tested offline on another run (and vice versa). In these paradigms, the tactile modality was clearly superior to other modalities, displaying high offline accuracy even when classification was performed on single trials only. Consequently, we tested the tactile paradigm online and the patient successfully selected targets without any error. Furthermore, we investigated use of the visual or tactile modality for different BCI systems with more than two selection options. In the visual modality, several BCI paradigms were tested offline. Neither matrix-based nor so-called gaze-independent paradigms constituted a means of control. These results may thus question the gaze-independence of current gaze-independent approaches to BCI. A tactile four-choice BCI resulted in high offline classification accuracies. Yet, online use raised various issues. Although performance was clearly above chance, practical daily life use appeared unlikely when compared to other communication approaches (e.g., partner scanning). Our results emphasize the need for user-centered design in BCI development including identification of the best stimulus modality for a particular user. Finally, the paper discusses feasibility of EEG-based BCI systems for patients in classic locked-in state and compares BCI to other AT solutions that we also tested during the study.

## Introduction

Damages to neuromuscular pathways, e.g., due to a stroke in the brainstem, or neurodegenerative diseases such as amyotrophic lateral sclerosis (ALS) or spinal muscular atrophy (SMA) can entail a severe loss of voluntary muscular control. These patients are summarized under the term locked-in syndrome (LIS; Plum and Posner, 1966) as they are locked into their own body despite often intact cognitive functioning. Patients in classic LIS are in total paralysis except for retaining control of vertical eye movements (Bauer et al., 1979). Consequently, communication is severely restricted for patients in this state. They usually rely on communication partners and utilize remaining eye muscle control (blinking or moving eye-brows) to answer questions in the closed format (yes/no) or to select suggested options.

Brain-computer interfaces (BCIs) were proposed as an alternative communication channel bypassing the requirement for retaining muscular control (for review, e.g., Kübler et al., 2001; Birbaumer and Cohen, 2007; Birbaumer et al., 2008; Allison et al., 2012; Wolpaw and Wolpaw, 2012). BCIs based on classification of event-related potentials (ERP) in the electroencephalogram (EEG) of a patient are most frequently used for communication purpose (Farwell and Donchin, 1988; for review, e.g., Kleih et al., 2011; Mak et al., 2011; Sellers et al., 2012). Several options (e.g., characters for typing words) are iteratively presented and users focus their attention on presentation of the one option they intend to select. Such target stimuli will elicit more pronounced ERPs than all other, irrelevant non-target stimuli. The procedure is thus referred to as oddball paradigm, as the target stimulus is rare compared to the frequent occurrence of irrelevant, non-target stimuli. ERP–BCIs usually rely highly on the P300 component (Sutton et al., 1965), a positive potential deflection occurring in the period of 200–500 ms post-stimulus (for review, Polich, 2007). Importantly, the P300 can be elicited in different modalities, i.e., visually, auditory or tactually. Thus, ERP–BCIs relying on any of the three modalities have been introduced (visual, Farwell and Donchin, 1988; auditory, Hill et al., 2005; Sellers and Donchin, 2006; tactile, Aloise et al., 2007; Brouwer and Van Erp, 2010; for review, Riccio et al., 2012).

Visual ERP–BCIs present characters for typing (or other selection options) on a screen. Usually, they are arranged in a matrix so that groups of characters can be stimulated at once, e.g., row/column wise (Farwell and Donchin, 1988; for review, e.g., Sellers et al., 2012). Stimulation can be performed by highlighting characters (i.e., light-flashing; Farwell and Donchin, 1988) or, as recently proposed, by overlaying them with faces (e.g., Kaufmann et al., 2011, 2013a; Zhang et al., 2012; Jin et al., 2012). However, matrix based ERP–BCIs may require accurate gaze control, therefore limiting its feasibility for people with LIS (Brunner et al., 2010; Treder and Blankertz, 2010). Thus, so-called gaze-independent paradigms have been suggested that present characters in the center of the screen (e.g., Acqualagna et al., 2010; Treder and Blankertz, 2010; Liu et al., 2011; Aloise et al., 2012; Acqualagna and Blankertz, 2013).

Auditory ERP–BCIs present sound stimuli that may differ in terms of volume, pitch, direction or combinations of those (e.g., Hill et al., 2005; Halder et al., 2010; Höhne et al., 2010, 2011; Schreuder et al., 2010, 2011a, 2013; Käthner et al., 2013) or differ with regard to informational content (e.g., Sellers and Donchin, 2006; Furdea et al., 2009; Klobassa et al., 2009; Kübler et al., 2009). Furthermore, stimuli may be presented sequentially or as a continuous stream (e.g., Hill and Schölkopf, 2012).

Tactile ERP–BCIs utilize stimulation units (further referred to as tactors; e.g., vibration motors or piezo elements) placed at different body locations, e.g., on hands, around the waist or on the back of participants (e.g., Aloise et al., 2007; Brouwer and Van Erp, 2010; Brouwer et al., 2010; Thurlings et al., 2012; van der Waal et al., 2012; Kaufmann et al., 2013b). Users focus their attention on tactile stimulation of one location they intend to select (target stimulus) and ignore stimuli on all other locations.

Aloise et al. (2007) compared the modalities in terms of achieved classification accuracies of eight participants. Results yielded strong superiority of the visual modality in terms of higher ERP amplitudes and lower latencies, thus enhancing classification accuracy. Consequently, all participants achieved best accuracy with visual stimulation, except for one participant who achieved equal performance in tactile and visual modality. However, this study faces the limitation that all participants were healthy. As described above, patients in classic LIS have impaired vision and thus results may differ.

Herein we report a case study with a locked-in patient for who we aimed at developing a BCI for communication. To our knowledge, this is the first report on tactile ERP classification in a patient with classic LIS. We compared ERPs evoked in all three modalities, investigated reliability of classification and further explored issues involved in the use of visual paradigms. Finally, we emphasize the requirement for user-centered design in BCI development and discuss limitations of current EEG-based BCI systems compared to other assistive technology (AT).

## Materials and methods

### The case

We visited a 46-year-old Italian woman twice for intensive testing on 7 days in total (first visit: 4 days; second visit: 3 days; with morning and afternoon testing sessions on most days). She had a brainstem stroke in the pons 7 years ago and since then has been in the classic locked-in state (for definition: Bauer et al., 1979). As confirmed by computed tomography (CT), the lesion barely affected her cortical abilities and she was fully attentive during all testing sessions. During the last year, she has been regaining some (still unreliable) control of her right thumb. Still reliable communication is only possible with vertical eye movements (partner scanning). As she cannot well accommodate, her left eye was partially sutured to avoid double vision. With her right eye fixation was possible, but never for more than few seconds and thus, she had to re-focus constantly.

**Figure 1:**
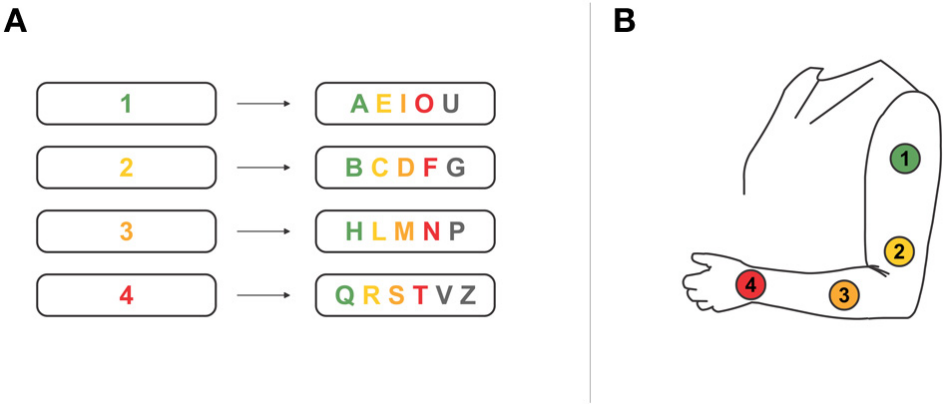
**(A)** Spelling system used by the patient in a partner scanning approach. Characters are grouped into four categories to increase spelling speed. **(B)** We developed a BCI system based on the partner scanning approach described in **(A)**. Four tactile stimulation units were placed on the patient's left arm. We individually adjusted the BCI paradigm to the partner scanning approach the patient is used to. Each tactor either represented one of the groups of characters, or represented a character of one prior selected group. Please note that we restricted the number of tactors to four, thus limiting the possible selections. Practical use of the system would require seven tactors (up to six tactors for selection of characters plus one tactor to undo a wrong selection).

She currently has no means of independent communication, i.e., communication is only possible in a partner scanning approach. Her dialog partner suggests letters or statements in the closed format that she can either select/agree with (eyelift) or not select/disagree (looking down). To enhance communication speed the patient utilizes an interval approach, i.e., characters were sorted according to their importance in Italian daily language and grouped into four categories (Figure 1A). First, the dialog partner reads out the categories (“first, second, third, fourth”) and she selects one category. Next, the letters of the selected category are read out and again she makes a choice (“A, E, I, O, U” for the first category; “B, C, D, F, G” for the second; “H, L, M, N, P” for the third and “Q, R, S, T, V, Z” for the fourth category).

The patient rated her quality of life as indexed by the *ACSA* [Anamnestic Comparative Self-Assessment Scale for Measuring the Subjective Quality of Life; scale from -5 (worst time in life) to 5 (best time in life); Bernheim and Buyse, 1993] as the worst time in her life (ACSA = −5). Asked for the reason, she answered “Desperate because I depend on others and I do not see a solution.” For 1 week prior to our first visit, caregivers daily assessed her mood and health as well as satisfaction with communication and nursing using questionnaires (linear scales from 0 [extremely bad] to 10 [excellent]). She rated mood low to medium (*M* = 5.0, range 3–6) and health as slightly above medium (*M* = 5.6, range 5–7). She had a cold prior to our first visit with fits of coughing leading to spasms. She was satisfied with nursing (*M* = 7.2, range 7–8) but displayed greater variance with regard to satisfaction with communication (*M* = 5.8, range 2–7). During our first stay, she reported one incidence where she had physical pain but was not able to call attention due to the absence of a communication partner. Establishing an independent communication ability is thus of utmost importance for her.

Prior to all testing sessions, we verbally informed the patient in detail about the procedure and obtained her consent to participate in the study through partner scanning. As no means of independent communication was possible, we asked her prior to every run if she agreed to proceed. The experiment was conducted in accordance with standard ethical guidelines as defined by the Declaration of Helsinki (World Medical Association) and the European Council's Convention for the Protection of Human Rights and Dignity of the Human Being with regard to the Application of Biology and Medicine (Convention on Human Rights and Biomedicine).

We approached the intended development of a BCI-based communication channel from a user-centered perspective targeting an individually tailored BCI solution for the patient. (1) We presented her with several classic oddball paradigms in three different modalities to identify the most promising modality for BCI use. (2) We tested different settings of BCI paradigms to explore emerging issues related to, e.g., system timing, gaze requirement, modality. Apart from BCI paradigms, we also tested other AT.

### Experimental design

**Figure 2:**
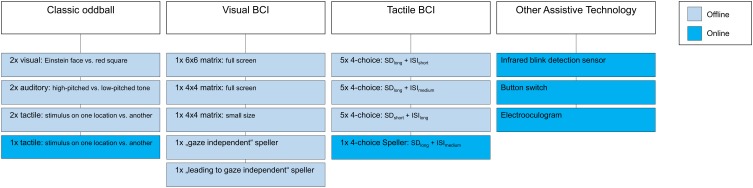
**Illustration of paradigms and systems tested during the visits**.

Figure 2 illustrates the various different paradigms that were tested during the visits.

#### Classic oddball paradigms

**Figure 3:**
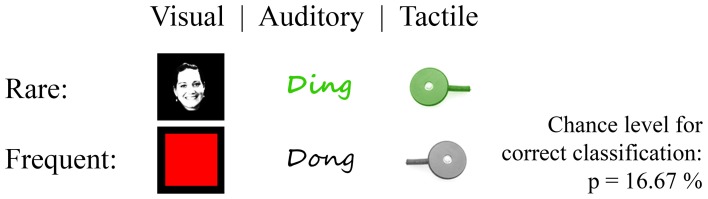
**Classic oddball paradigms in three modalities.** Stimuli where Einstein face vs. red square displayed in the center of a black screen (visual; the figure exemplarily displays another face due to printing license), high vs. low pitched tone (auditory) and tactile stimulation at one position vs. stimulation on a second position (tactile). Rare stimuli to frequent stimuli ratio was 1:5.

All tested oddball paradigms shared the same parameters except for the modality and stimulus duration (SD) (Figure 3). Two stimuli were presented with an inter-SD of 1000 ms and a rare to frequent ratio of 1:5. One run comprised 90 rare and 450 frequent stimuli. We conducted two runs per modality.

***Offline classification***. To investigate reliability of each modality, we build weights of a stepwise linear discriminant analysis (SWLDA; e.g., Donchin, 1969; Farwell and Donchin, 1988; Krusienski et al., 2006) based on one of two runs and tested the classifier on the other run (and vice versa). We used 1000 ms of data post-stimulus for classification. In ERP–BCIs, higher reliability is usually achieved by considering several trials for classification. To align with this setting we grouped trials into several blocks for offline classification. This led to classification based on 15 trials, based on 5 trials, 3 trials, 2 trials and finally to classification of single trials respectively.

***Online classification***. Apart from the above described offline classification we conducted one run with online classification in the tactile modality. A classifier was built from the two classic tactile oddball runs. The task was to select the target stimulus four times and online classification was performed each time after 20 rare and 100 frequent stimuli. Feedback was immediately presented to the patient in that we verbally communicated classification outcome.

***Visual oddball***. A red square of size 100 × 100 pixels was displayed frequently in the center of a screen with black background. The odd stimuli were of same size and displayed the famous picture of Albert Einstein presenting his tongue. We modified the picture in that it only displayed the face on a black background. SD was 64.25 ms.

***Auditory oddball***. Stimuli were presented with headphones to both ears with same volume for all stimuli. SD was 400 ms. Odd stimuli comprised a high-pitched tone whereas irrelevant stimuli comprised a low-pitched tone.

***Tactile oddball***. Target and non-target stimuli did not vary in terms of SD (220 ms), vibration frequency or vibration gain, but only with respect to the location. The task was to focus attention on one location while ignoring stimuli on the other. To account for sensitivity differences on two forearm locations, we switched target and non-target location between the two runs. As such, we made sure that elicited ERPs following rare stimuli are not due to decreased sensory perception capabilities on the location of frequent stimuli.

#### Visual BCI

The patient reported to see the entire screen placed approximately 80–90 cm distant to her. We pointed to different locations in a visually displayed character matrix, in particular to the corners, and asked if she could see these locations (closed question by means of partner scanning; “Can you see the character displayed at this location?”). The tested visual matrices were of different size (large matrix grid on full screen; smaller matrix grid in the center of the screen), contents (6 × 6 matrix; 4 × 4 matrix) and timing [short, medium and long inter-stimulus interval (ISI)]. We adjusted the latter settings based on the patient's report after each testing run. Stimuli in all matrix paradigms comprised the famous face of Albert Einstein as introduced earlier (Kaufmann et al., 2011, 2013a), i.e., the famous face overlaid characters (face flash) and the patient counted the number of face flashes on top of the intended character. We explicitly told the patient to focus attention continuously on face flashes on top of the target character even if she was not able to keep her gaze focused on the target.

Apart from matrix paradigms, we tested a so-called gaze-independent paradigm in which characters were presented consecutively in the center of the screen. We used only six characters (A–F) to align with the properties of the visual oddball paradigm. The target character was the “D.” Furthermore, we tested one setting, to bridge between the oddball paradigm and the gaze-independent speller. Instead of the “D,” it displayed the Einstein face as for the visual oddball paradigm. Yet, in contrast to the visual oddball, frequent stimuli comprised characters instead of the red square (thus referred to as “leading to” gaze-independent speller).

#### Tactile BCI

We checked sensory sensitivity of the patient's left forearm and upper arm by stimulating different locations and inquiring her perception capabilities (two closed questions by means of partner scanning; “Do you feel the stimulus?”; “Do you feel the stimuli approximately equally well?”). Four tactors (see section Equipment, Data Acquisition and Analysis) were then placed with around 10–15 cm distance on her left forearm and upper arm (see Figure 1B).

We investigated different timing parameters in several sessions to define the setting in which discrimination of four tactors would work best for the patient. Each setting comprised five runs, each run with every tactor being the target once. (1) SD was long (520 ms) and ISI was short (200 ms). Each tactor was stimulated 15 times per selection, resulting in 60 target and 180 non-target stimuli per run. (2) SD was long (520 ms) and ISI was medium (520 ms) with again every tactor being stimulated 15 times per target. (3) SD was short (220 ms) and ISI was long (800 ms). We increased the number of stimulations by factor 2 to gather more data, resulting in 120 target and 360 non-target stimuli. For direct comparison of the first and the second setting, we reduced the number of stimulations in the offline analysis.

For comparison of these settings, we trained SWLDA classifiers on every combination of four of five runs and tested classification outcome on the remaining run (see first three columns of Table 1 for illustration of all combinations). Furthermore, we assessed classification outcome for classifier weights trained on 800 ms of data, 1000 ms, 1200 ms, and 1400 ms respectively.

**Table 1:** **Classification accuracy based on different runs of tactile BCI use**.

**Data set**	**Classifier trained on runs #**	**Classifier tested on runs #**	**800 ms post-stimulus**	**1000 ms post-stimulus**	**1200 ms post-stimulus**	**1400 ms post-stimulus**
Long stimulus, short ISI	[1 2 3 4]	[5]	50	75	75	75
	[1 2 3 5]	[4]	25	25	75	100
	[1 2 4 5]	[3]	50	75	100	75
	[1 3 4 5]	[2]	100	100	100	100
	[2 3 4 5]	[1]	50	100	100	100
	Mean ± STD		55.5 ± 27.4	75.0 ± 30.6	90.0 ± 13.7	90.0 ± 13.7
Long stimulus, medium ISI	[1 2 3 4]	[5]	75	100	100	100
	[1 2 3 5]	[4]	75	50	100	100
	[1 2 4 5]	[3]	50	50	75	50
	[1 3 4 5]	[2]	0	0	50	25
	[2 3 4 5]	[1]	50	50	75	50
	Mean ± STD		50 ± 30.6	50 ± 35.4	80 ± 20.9	65 ± 33.5
Short stimulus, long ISI. Number of stimulations reduced offline for direct comparison	[1 2 3 4]	[5]	75	75	50	75
	[1 2 3 5]	[4]	75	50	75	100
	[1 2 4 5]	[3]	75	75	75	75
	[1 3 4 5]	[2]	25	75	50	0
	[2 3 4 5]	[1]	75	75	75	75
	Mean ± STD		65 ± 22.4	70 ± 11.2	65 ± 13.4	65 ± 37.9
Short stimulus, long ISI. Full data set with twice as much stimuli.	[1 2 3 4]	[5]	100	75	100	100
	[1 2 3 5]	[4]	75	75	75	100
	[1 2 4 5]	[3]	75	75	75	75
	[1 3 4 5]	[2]	75	75	75	100
	[2 3 4 5]	[1]	75	75	50	50
	Mean ± STD		80.0 ± 11.2	75.0 ± 0.0	75.0 ± 17.7	85.0 ± 22.4

We trained SWLDA classifiers on every combination of four of five runs and tested them on the remaining run. The table furthermore presents classification outcome for classifier weights trained on 800 ms of data, 1000 ms, 1200 ms, and 1400 ms respectively.

Finally, we conducted one run, in which the patient used the tactile BCI for communication. We implemented a BCI spelling system analog to her partner scanning approach (see Figure 1). First, one of four groups of characters was selected, followed by selection of an individual character. As our setup and calibration was restricted to a four-choice paradigm, we only enabled selection of the first four characters in each group for this online test (colored characters in Figure 1). The patient tested this system in one run aiming at copy spelling a four-letter word (8 selections, i.e., four times selection of group plus four times selection of character). SD was the same as ISI duration, both 520 ms long.

#### Other assistive technology

In this study, we investigated feasibility of a BCI system as a communication channel alternative to the partner scanning that she currently uses. Yet, during the visits, we also attempted to provide her with other AT as no reliable communication method other than partner scanning has ever been established within the past 7 years. During our first visit we tried two commercial AT devices, (1) an infrared blink detection sensor (*SCATIR*, Prentke Romich GmbH) and (2) a button (*Lib Switch*, Prentke Romich GmbH) on her thumb. We connected it to a communication device that allows for selecting characters or commands (*XLTalker*, Prentke Romich GmbH). During our second visit, we investigated use of an electrooculogram (EOG) for detection of eyelifts. We connected one EOG electrode placed below her right eye to our BCI software and classified eyelifts using SWLDA. The software read out characters in the same manner as the above-described partner scanning approach the patient is used to (see also section Tactile BCI and Figure 1A). When the software read out the intended group or the intended character respectively, the patient lifted her eyebrow thereby triggering a reliable deflection in the recorded muscle activity.

### Equipment, data acquisition, and analysis

Visual stimulation was performed on a 22″ screen (LG Flatron; 1680 × 1050 pixels), auditory stimulation through headphones fully covering both ears (Sennheiser, HD280 pro) and tactile stimulation with small vibrate transducers (C2 tactors; Engineering Acoustics Inc., USA). We implemented the stimulation paradigms for all modalities in Python 2.7 (www.python.org) and connected them to the BCI2000 software (Schalk et al., 2004; www.bci2000.org) via user datagram protocol.

EEG during oddball-paradigms was obtained from 11 passive Ag/AgCl electrodes with mastoid ground and reference placed at positions Fz, FC1, FC2, C3, Cz, C4, PO7, P3, Pz, P4 and PO8. For testing BCI paradigms, we extended the electrode setup by four electrodes (TP7, CP3, CP4 and TP8) to a 15 electrodes setting. EEG was amplified with a g.USBamp amplifier (g.Tec Medical GmbH, Austria) and recorded at 512 Hz using BCI2000. Data was analyzed in Matlab 2012 (The Mathworks Inc., USA) and classification of oddball paradigms as well as all BCI paradigms performed utilizing SWLDA (e.g., Donchin, 1969; Farwell and Donchin, 1988; Krusienski et al., 2006).

## Results

### Classic oddball paradigms

**Figure 4:**
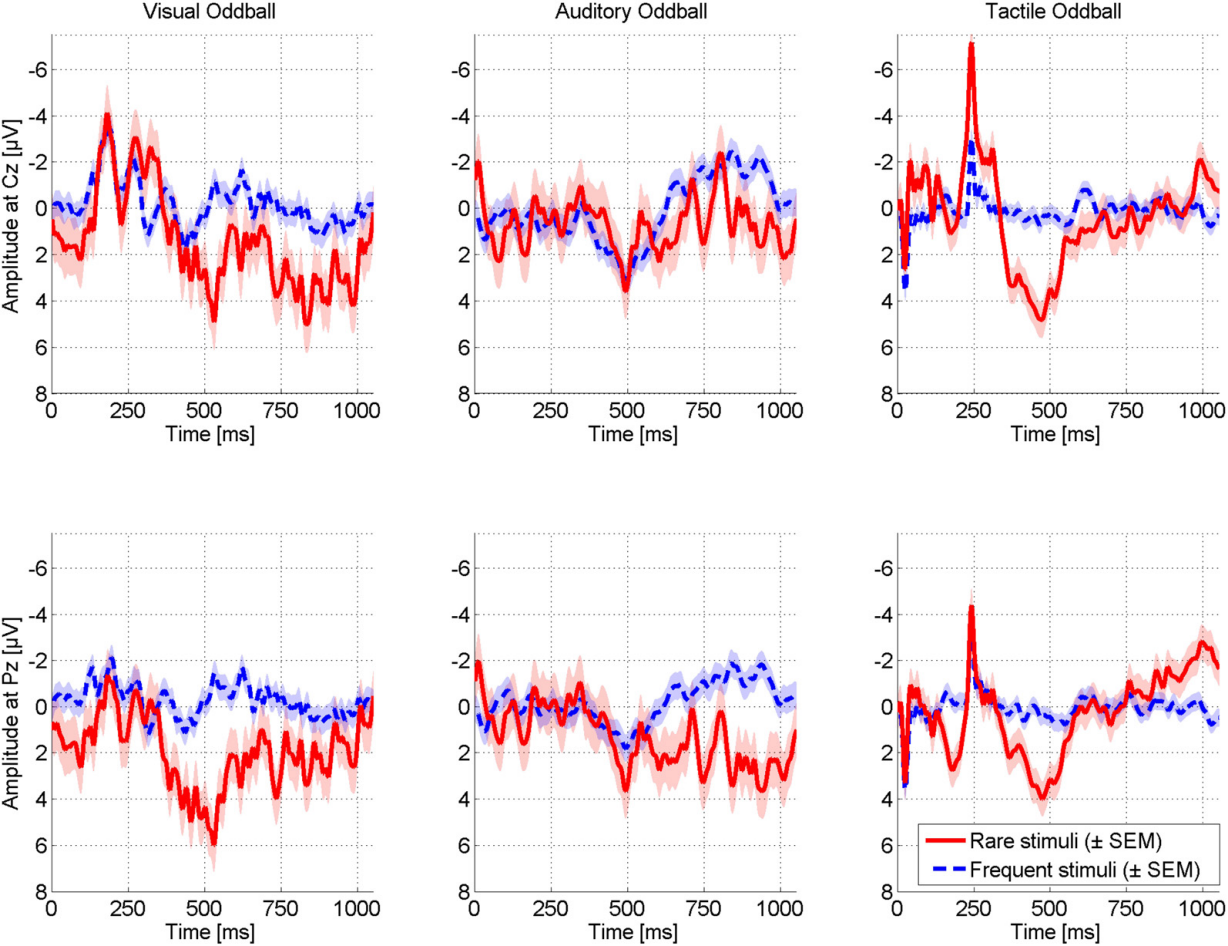
**Comparison of ERPs elicited in different modalities in the classic oddball paradigms.** ERPs are exemplarily displayed for electrode Cz (upper row) and Pz (lower row). Visual and tactile stimulation elicited the most pronounced differences between target and non-target stimulations. Reliability across trials was highest for the tactile modality (see Figure 5).

Figure 4 displays ERPs elicited in the oddball paradigms for two exemplary electrodes. Difference between rare and frequent stimuli was most pronounced for visual and tactile modalities displaying a distinct P300 around 500 ms post-stimulus. Peak amplitudes were of same size for the visual (5.96 μV, 530 ms, Pz) and the tactile modality (5.92 μV, 471 ms, FC2) and higher than for the auditory modality (3.95 μV, 493 ms, Fz).

**Figure 5:**
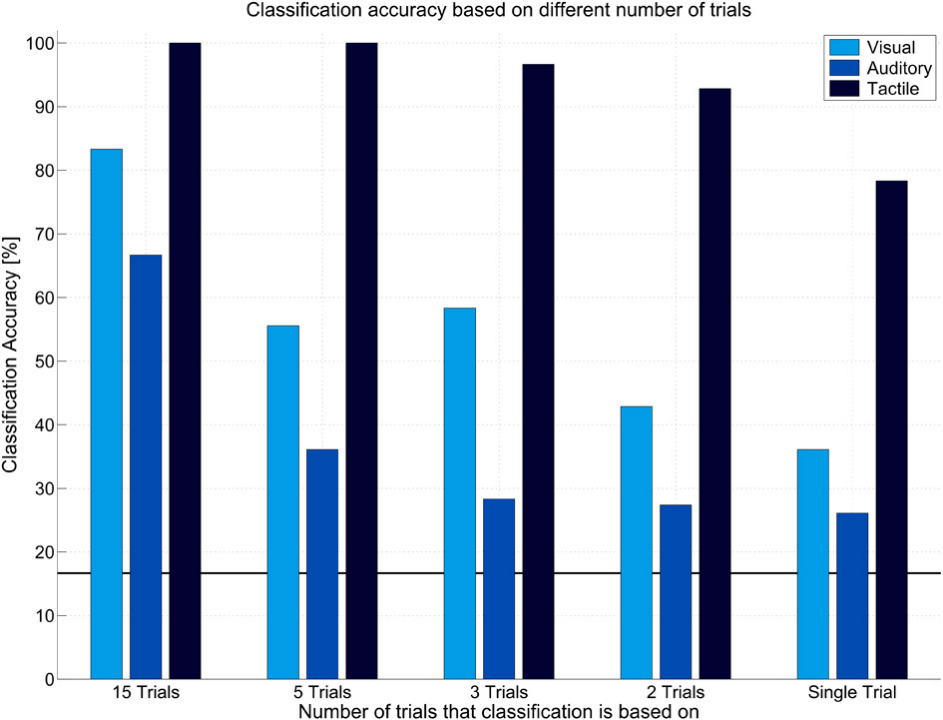
**Offline classification accuracy achieved in different modalities in the classic oddball paradigms.** Classification accuracies are presented based on classification of single trials, two, three, five, or fifteen trials. Tactile modality outperformed other modalities in that high classification accuracy could be achieved even based on single trials.

To investigate the reliability of elicited ERPs offline, we trained classifiers for each modality based on one run and tested them on the other run (and vice versa). Figure 5 depicts average offline classification accuracies. The tactile modality was clearly superior to the visual and auditory modality. Although classification of visual and auditory ERPs was possible when including many trials into classification (visual: *M* = 83.33%, auditory: *M* = 66.67% with 15 trials), performance severely decreased with reduced number of trials. This effect was more pronounced in the auditory modality. Classification accuracy based on few trials was insufficiently low (below *M* = 60% with 3 trials or less). In contrast, in the tactile modality five or more trials led to 100% classification accuracy. Importantly, accuracy was still high if based on two trials (*M* = 92.85%) and even if based on single trial (*M* = 78.33%).

We conducted an online test session with the tactile oddball paradigm. The patient correctly selected the target in all cases, i.e., online classification accuracy based on 10-trials of two tactile stimuli was 100%.

### Transfer to BCI

As visual and tactile oddballs displayed pronounced ERPs post-stimulus, we tested these modalities with BCI paradigms.

#### Visual BCI

**Figure 6:**
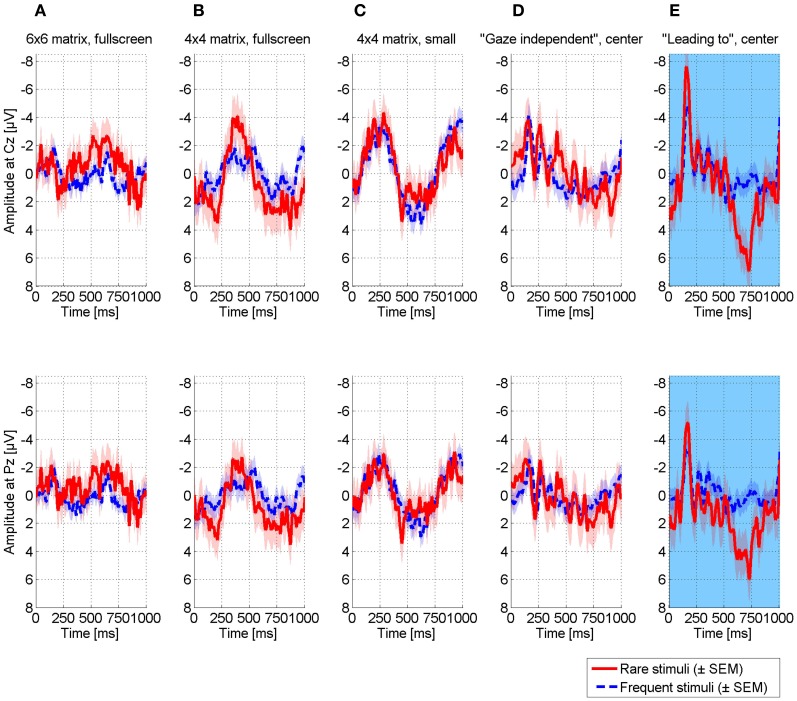
**Comparison of ERPs elicited in different visual BCI paradigms, exemplarily illustrated for electrode Cz (upper row) and Pz (lower row). (A)** 6 × 6 matrix presented in fullscreen, **(B)** 4×4 matrix presented in fullscreen, **(C)** 4×4 matrix presented at smaller size in the center of the screen, **(D)** “Gaze independent” speller, characters were presented in the center of the screen **(E)** “Leading to” gaze independent speller, similar to the gaze independent speller except for the target stimulus that was replaced by a face stimulus. Neither matrix based paradigms **(A-C)**, nor a gaze-independent paradigm **(D)** led to reliable differences between target and non-target stimulations. To investigate potential sources why the gaze-independent paradigm did not work, we conducted one run in which the target character was replaced with a face **(E)**. This paradigm is similar to the visual oddball and differed only with regard to the non-target stimuli. As this paradigm elicited pronounced ERPs that compared to the visual oddball, we assume that identification of a character in a plethora of presented characters may be aggravated and sufficient gaze control may be required.

Although the patient reported to perceive the entire screen (see section Experimental Design), matrix-based BCI paradigms were not viable. After initial testing with a 6 × 6 matrix, we reduced the number of matrix items to 4 × 4 and finally we reduced the size from full-screen to a small matrix in the center of the screen. Yet, none of these paradigms evoked pronounced and thus, reliably classifiable ERPs (Figures 6A–C). No N170 was visible as would have been expected if recognizing the face presented on the target character (Bentin et al., 1996; Eimer, 2000). The patient reported difficulties in continuously focusing on a target, although possible for a short time.

As she had trouble with focusing her gaze on targets, we tested a so-called gaze-independent BCI paradigm, randomly presenting six characters in the center of the screen. However, also this paradigm failed such that no reliable ERPs were elicited (Figure 6D). As the visual oddball elicited pronounced ERPs, we further investigated possible reasons for failure of the gaze-independent paradigm. In the visual oddball, the black and white Einstein face was easily distinguishable from the red squares. Thus, we combined the oddball with the gaze-independent speller such that five white characters were used as non-targets and the (black and white) Einstein face as target. As depicted in Figure 6E, the paradigm elicited pronounced ERPs including a strong N170. The P300 was even of higher amplitude compared to the visual oddball (7.78 μV, Fz), however, its latency was strongly increased (723 ms), indicating increased difficulty to discriminate between target and non-target stimuli (Figure 4).

#### Tactile BCI

**Figure 7:**
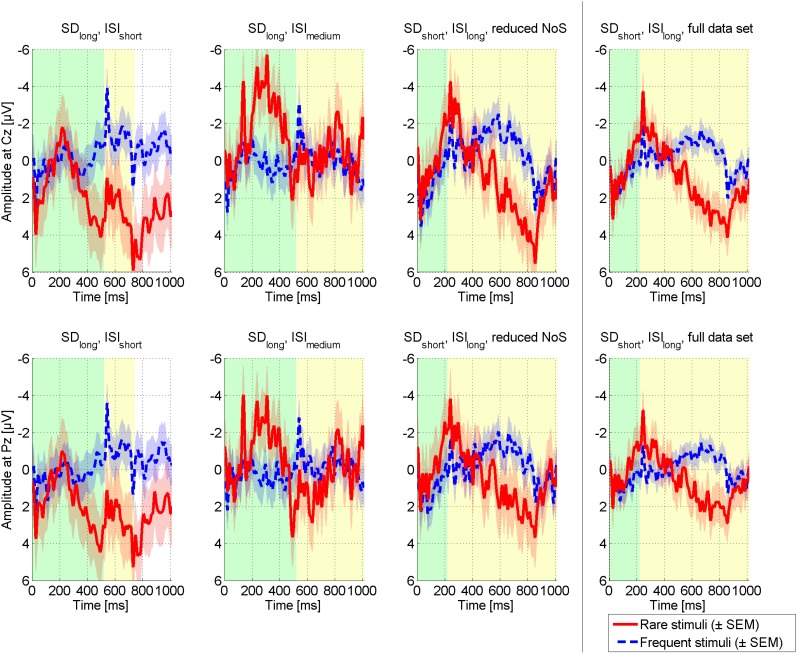
**Comparison of ERPs elicited in a four-choice tactile BCI with different stimulus parameters.** Stimulus duration (SD) was either long (520 ms) or short (220 ms). Inter-stimulus interval (ISI) was short (200 ms), medium (520 ms) or long (800 ms). Three combinations of SD and ISI were tested exploratory, i.e., (1) SD_long_ + ISI_short_, (2) SD_long_ + ISI_medium_, and (3) SD_short_ + ISI_long_. Please note that for the third combination, more data was available. For comparison, we thus reduced the amount of data to similar size for all conditions. Yet, the full data set is depicted in the plot on the right.

As the visual modality appeared unreliable, we further focused on the tactile modality. We extended the setup to a four-choice BCI paradigm and conducted 15 runs in total where each of four tactors was the target once per run. Figure 7 compares the ERPs elicited in different settings (long SD + short ISI; long SD + medium ISI; short SD + long ISI). The condition with long SD and short ISI elicited most pronounced ERPs, followed by the condition with short SD and long ISI. Importantly, ERPs of all conditions could be classified offline with high accuracies (see Table 1). In general classification on 1200 ms and 1400 ms post-stimulus achieved highest accuracy yet variance was lowest for classification based on 1200 ms. All obtained classification results were clearly above the chance level of 25%.

Finally, we tested a communication application online, utilizing the tactile four choice BCI described in section Tactile BCI. The patient completed one run with 50% online accuracy (four of eight selections correct).

### Other assistive technology

Communication by means of an infrared blink detection sensor failed due to the presence of involuntary muscle movements of her eyelids. Use of a simple button on her thumb appeared more promising. Although still unreliable, selections were clearly above chance.

The EOG based eye lift detection tested during our second visit appeared far most promising. After a short calibration of 8 min only, the patient could use the EOG for reliable communication and spelled several words without error. Classification was performed after every three trials of eye lifts. This system for the first time provided a reliable and fast means of independent communication.

## Discussion

This case study with a LIS patient revealed the potential of tactile stimulation for BCI use such that tactually evoked ERPs were clearly more reliable than those elicited in the visual or auditory modality. Although an average across 180 target trials per modality led to similar ERP amplitudes for the tactile and the visual domain, visual ERPs were much less reliable. With single-trial offline classification of tactile ERPs in the oddball paradigm, almost the same level of classification accuracy was obtained (*M* = 78.33) as with 15 trials of classification in the visual modality (*M* = 83.33). These promising offline results were replicated in an online run in which the patient correctly selected the target stimuli without any error (four times, each based on 20 rare and 100 frequent tactile ERP stimuli).

When extending the setting to four tactile stimulation units, ERPs of same amplitude were elicited. Classification accuracies as depicted in Table 1 were up to 100% and for all settings clearly above chance level. Performance achieved in runs based on long SD and short ISI was higher than in other settings (*M* = 90%). ERPs depicted in Figure 7 render a short SD feasible for tactile ERP elicitation in our patient, yet classification accuracy was not as high. The larger SD may have increased the patient's stimulus perception ability. Brouwer and Van Erp (2010) reported significantly decreased classification accuracy for a condition with long SD (367 ms) and no ISI (0 ms). Our results complement these findings in that the decreased performance may not be due to the increased SD but due to the missing ISI. The authors further reported, that for a condition with sufficiently long SD (188 cms), performance could be further increased by decreasing the ISI (SD: 188 ms, ISI: 188 ms). This finding is in line with our results, where a shorter ISI entailed better accuracy than a longer ISI.

In the test of the tactile spelling system, the patient achieved an accuracy of 50% only. Although this result was above chance, it is insufficient for communication (Kübler et al., 2001). Choice of 520 ms SD and 520 ms ISI might have been suboptimal when considering the results from the comprehensive offline analysis conducted afterwards (depicted in Table 1). Consequently, we expect higher accuracies for future tests, when applying a shorter ISI. Also, the patient had a strong cough during the last character selection process, explaining the last miss-selection. As noted by a family member, these coughs particularly appear when she is excited and endeavored (see also section General Implications with EEG-Based BCIs and Comparison to Other Assistive Technology). To use the proposed spelling system in full functionality, an extension from four to seven tactors would be required. Brouwer and Van Erp (2010) reported similar classification accuracies when using two, four, or six tactors. Our results yielded decreased performance when extending the setup from two to four tactors. Although accuracies were still high, they were lower in the four-choice tactile BCI than expected from the classic oddball paradigm results. If an extension to seven tactors may be feasible for the patient remains to be investigated.

Notwithstanding these caveats, our results were a proof of concept for the feasibility of tactile stimulation for BCI control in a patient for who the visual modality did not work in any setting.

Apart from these promising results on tactually evoked ERPs, we reported on two other modalities. The auditory modality appears least promising in this patient. ERP amplitudes were lower as compared to other modalities and offline classification accuracy rapidly decreased when reducing the number of trials. Here we used stimuli that varied in pitch as a study by Halder et al. (2010) reported such stimuli superior to those that varied in volume or direction. Yet other differentiations between rare and frequent stimuli may yield better results, e.g., combinations of pitch and spatial information (Schreuder et al., 2010; Höhne et al., 2011; Käthner et al., 2013). Recently, Halder et al. (2013) illustrated that training may positively affect auditory BCI performance. Furthermore, as for tactile BCIs, SD may strongly affect classification outcome. No definite conclusion can thus be drawn for the auditory modality.

For the visual modality, we conducted many runs in different settings and were thus able to draw a more detailed picture than for the auditory modality. Matrix-based visual ERP–BCIs failed in all of the tested configurations. Although able to see the entire screen, the patient had difficulties in focusing for a longer time on a peripheral location. However, it is notable that a so-called gaze-independent speller did not work either. Focusing attention on the target character seemed not sufficient for correct selection. A possible explanation is that these paradigms in fact do require gaze control for discrimination between characters. To investigate this hypotheses we compared ERPs elicited in the visual oddball (Einstein face vs. red squares) to ERPs elicited in a paradigm with the black and white Einstein face as target and white characters as non-targets. This paradigm elicited a strong P300, yet the peak was delayed as compared to the visual oddball. This delay may be due to an increased difficulty in discriminating targets from non-targets. Consequently, we assume that enhancing discriminability between characters may entail better results in her case. Acqualagna et al. (2010) compared a condition in which characters were presented in different colors to a condition with black characters only. Participants achieved better counting accuracy when characters were of different color, yet offline classification was lower compared to the black-character condition. In the follow-up online study classification accuracy was the same for both conditions (Acqualagna and Blankertz, 2013). Thus, we would not expect a boost in performance from such modification. Other “gaze-independent” spellers could be tested, e.g., a speller that groups characters into categories (Treder and Blankertz, 2010). However, from the classification accuracy achieved in the visual oddball we would not expect reliable communication based on the visual modality.

Our results manifest the importance of user-centered design in BCI development (Maguire, 1998; Zickler et al., 2011; Holz et al., 2012). Based on data obtained from healthy participants, we expected the visual modality (gaze-independent) to be superior to the others (e.g., the direct comparison of modalities by Aloise et al., 2007; accuracies reported from studies conducted in different modalities, for review, Riccio et al., 2012). Clearly, this was not the case in our patient, which convincingly demonstrates that results achieved with healthy subjects do not necessarily transfer to locked-in patients. BCIs that yield lower results in healthy users may be the only possible setting for a particular end-user with motor impairment or in the locked-in state. Thus, when aiming at bringing BCIs to end-users, those have to be included in the developmental process for which the user-centered design provides a framework (Maguire, 1998; Holz et al., 2012). Only when specifically investigating the requirements of a targeted end-user a well-suited BCI can be implemented as their needs and requirements may well differ from that of healthy users (Zickler et al., 2011). In addition, development of a BCI system that copies the communication approach a patient is used to may increase learnability of system control. The approach we implemented in this study (Figure 1) was similar to the patient's approach, which she highly appreciated.

In our case study, the patient rated her perceived quality of life as “the worst time in life” (see section The Case) and explained her low rating as being due to the strong dependence on others. In a survey among 65 LIS patients, Bruno et al. (2011) reported that only 28% of patients perceived unhappiness (*ACSA* scores below 0) and only 1/3 of them rated quality of life with an *ACSA* score of -5. Yet unhappiness was associated with non-recovery of speech production. These and our results manifest the importance of providing these patients with a means of independent communication.

### General implications with EEG-based BCIs and comparison to other assistive technology

Recent research demonstrated that independent BCI home-use by a locked-in patient is possible (Sellers et al., 2010; Holz et al., 2013) and that the software can be automatized such that naïve users can handle it (Kaufmann et al., 2012a). However, several issues remain, e.g., related to artifact contamination of EEG data or attention allocation capacity. Some of these issues may be:

1. Spasm artifacts: During our first visit, the patient had several spasm attacks (due to cough, see section The Case) so that we had to cancel runs and start again. On the last day, the attacks were so intense, that the BCI session had to be terminated. Apart from health related attacks, the patient also had coughs due to an increased excitement and endeavor (as noted by a family member). Future research should thus investigate algorithms for identification of artifacts from the ongoing EEG. A practical BCI should automatically pause in the case of too noisy EEG and proceed once artifact induced electrode drifts diminished. In addition, the EEG could be cleaned prior to computing classifier weights to avoid building classifiers based on artifacts. Furthermore, classification based on a dynamically adjusting number of trials may compensate small artifact contamination such that more trials can be presented if artifacts lower classification certainty (e.g., Lenhardt et al., 2008; Höhne et al., 2010; Liu et al., 2010; Jin et al., 2011; Schreuder et al., 2011a; for review, Schreuder et al., 2011b, 2013).
2. Single electrode drifts and cap displacement: Apart from spasm artifacts, that usually contaminate all electrodes, single electrode drifts or shift of cap placement should automatically be identified. During our stay, the patient had a strong spasm attack after which the whole electrode cap had shifted. Furthermore, single electrodes sometimes lost contact or even dropped out after such attacks. Dauce and Proix (2013) suggested a method for identification of performance drops during free spelling. The backspace key is used as an indicator of low performance. If used too frequently, the BCI is recalibrated.
3. Attention allocation and workload: In a home environment, background noise is present in daily life situations, e.g., phone rings, voice of others, etc. ERP–BCIs (especially non-visual ERP–BCIs) require high attention to stimuli (e.g., Kaufmann et al., 2012b) and such noise may badly affect performance. This clearly is a limitation compared to other assistive technologies. For example, the EOG based device that we provided to the patient (see section The Case) requires far less attention allocation and may thus prove more useful for her in daily life even if both systems would display equivalent bit rates.
4. Flexibility: In the partner scanning approach that the patient currently uses, the communication partner can easily repeat a scan if selection of a character was unclear or can even suggest another more likely character instead. Not only may these selections be based on the spelled characters but also be based on contextual knowledge of the patient's life. Furthermore, the partner will easily recognize if the patient was distracted. Consequently, the partner scanning approach is particularly flexible. First approaches to increase flexibility of BCI systems are available (e.g., the above described dynamic stopping, for review Schreuder et al., 2013; text prediction, e.g., Ryan et al., 2011; Kaufmann et al., 2012a; or error correction procedures, e.g., Dauce and Proix, 2013), yet compared to the partner scanning these systems still lack flexibility.
5. Evaluation: It is important to validate after each run if the patient could concentrate and if anything was disturbing or unclear. Although this is rather time consuming when considering that the patient can only communicate on a character-by-character basis in a partner scanning approach, it is a necessity. Otherwise, it is impossible to investigate if for example decreased performance results from a modification of the system or from decreased attention or distraction (Zickler et al., 2011, in press; Holz et al., 2013, in press).

Although we consider BCIs of particular interest for the patient described in this paper, the patient will not use a current BCI system for communication. As described in section The Case we tested an EOG based system that was far more reliable. However, the system requires muscular control which can be too fatiguing in frequent use. A tactile ERP–BCI would be a muscle-independent alternative. Importantly, the patient reported tactile BCI use as not being tiring. Thus, although we identified multiple issues that prevent transfer of current BCI technology to her daily life, the method should still be further explored as an alternative communication channel. If future research identifies solutions to the issues described above, BCIs may well be a feasible communication tool for patients in (classic) LIS - at least as a valuable alternative among other systems.

## Conclusion

This case study demonstrated successful classification of tactually evoked ERPs in a patient with classic LIS. The tactile modality was clearly superior to the visual and auditory modality. The patient achieved high accuracy even with a small number of trials in a two-class oddball and medium to high accuracy in a four-choice tactile BCI paradigm. Results from visual BCI paradigms may question gaze-independence of current gaze-independent spellers, as gaze-control may not only be required to focus peripheral targets but also for discrimination of characters presented in the center of the screen. Further, our results emphasize the need for user-centered design in BCI development and underline remaining issues when considering practical daily life use that are not as relevant with other assistive technologies.

### Conflict of interest statement

The authors declare that the research was conducted in the absence of any commercial or financial relationships that could be construed as a potential conflict of interest.
